# Quantifying the Treatment Effect of Kidney Transplantation Relative to Dialysis on Survival Time: New Results Based on Propensity Score Weighting and Longitudinal Observational Data from Sweden

**DOI:** 10.3390/ijerph17197318

**Published:** 2020-10-07

**Authors:** Ye Zhang, Ulf-G. Gerdtham, Helena Rydell, Johan Jarl

**Affiliations:** 1 School of Sociology and Population Studies, Renmin University of China, Beijing 100872, China; 2 Health Economics Unit, Department of Clinical Sciences, Malmö, Lund University, 22381 Lund, Sweden; ulf.gerdtham@med.lu.se (U.-G.G.); johan.jarl@med.lu.se (J.J.); 3 Department of Economics, Lund University, 22363 Lund, Sweden; 4 Centre for Economic Demography, Lund University, 22363 Lund, Sweden; 5 Department of Clinical Sciences Intervention and Technology, Karolinska Institute, 17177 Huddinge, Sweden; helena.rydell@sll.se; 6 Swedish Renal Registry, Department of Internal Medicine, Ryhov County Hospital, 55185 Jönköping, Sweden

**Keywords:** kidney transplantation, dialysis, inverse-probability-weighted regression adjustment approach, average treatment effect, survival time, Sweden

## Abstract

Using observational data to assess the treatment effects on outcomes of kidney transplantation relative to dialysis for patients on renal replacement therapy is challenging due to the non-random selection into treatment. This study applied the propensity score weighting approach in order to address the treatment selection bias of kidney transplantation on survival time compared with dialysis for patients on the waitlist. We included 2676 adult waitlisted patients who started renal replacement therapy in Sweden between 1 January 1995, and 31 December 2012. Weibull and logistic regression models were used for the outcome and treatment models, respectively. The potential outcome mean and the average treatment effect were estimated using an inverse-probability-weighted regression adjustment approach. The estimated survival times from start of renal replacement therapy were 23.1 years (95% confidence interval (CI): 21.2−25.0) and 9.3 years (95% CI: 7.8−10.8) for kidney transplantation and dialysis, respectively. The survival advantage of kidney transplantation compared with dialysis was estimated to 13.8 years (95% CI: 11.4−16.2). There was no significant difference in the survival advantage of transplantation between men and women. Controlling for possible immortality bias reduced the survival advantage to 9.1–9.9 years. Our results suggest that kidney transplantation substantially increases survival time compared with dialysis in Sweden and that this consequence of treatment is equally distributed over sex.

## 1. Introduction

Assessing the treatment effects of kidney transplantation (KTx) compared with dialysis using observational data from actual medical practice is the most feasible approach in the field of renal replacement therapy (RRT) for end-stage renal disease (ESRD) where randomized controlled trials (RCTs) are considered unethical [1]. However, observational data are subjected to treatment selection bias due to selection based on prognosis expectation (e.g., patients with a better prognosis are more likely to get kidney transplantation over dialysis) and the inability to adjust for all relevant patient characteristics [1]. The standard approach to the selection problem is to apply multivariable regressions although using a propensity score approach to adjust for selection bias has become increasingly popular [2].

In studies from the US [3,4] and in a Swedish single-center study [5] it has been shown that patients with a renal transplant have superior long-term survival compared to waitlisted patients on dialysis. Renal transplantation is more often considered as suitable for younger patients. However, studies from Australia [6] and Norway [7] have also shown that kidney transplantation seems to confer a survival advantage over dialysis in patients over 60 and 70 years, respectively. Sahar et al. [8] found that kidney transplantation was associated with improved survival compared to dialysis and that the benefit of kidney transplantation persisted among elderly patients (age ≥60 years). Miklos et al. [9] found that kidney transplantation was associated with improved survival compared to dialysis in elderly patients (age ≥65 years).

Observational studies have previously applied Cox regressions [3,4,6,7,10] and propensity score methods, separately or combined [8,9], in comparisons of the mortality for patients with a renal transplant or on dialysis. The common approach of comparing patients with kidney transplantation to patients on the waitlist may reduce the selection problem but it cannot completely control for selection bias as the patients that obtain a kidney transplant are generally younger and healthier [8]. Some previous studies applied Cox regression analyses combined with propensity score matching or stratification methods to reduce the selection bias for patients on the waitlist or for patients not limited by the waitlist [8,9]. However, studies [11,12] indicate that the propensity score weighting approach is the most general and most efficient because it uses all the available data and does not require any arbitrary decisions with regard to stratification on the propensity score or propensity score matching. In addition, the propensity score weighting method using the potential outcomes framework permits the estimation of both the relative and absolute survival in a treated population compared with an untreated population.

Therefore, this study uses the propensity score weighting approach for ESRD patients on the waitlist in order to estimate the potential outcome means (POMs) and the average treatment effects (ATEs) of kidney transplantation on survival time compared with dialysis within the Swedish healthcare system. Specifically, we will study: (1) whether kidney transplantation offers a survival advantage over dialysis and if so, by how much; and (2) whether the survival advantage differs over sex.

The current study contributes to the existing literature regarding the treatment effects of alternative RRT on survival time in at least three ways. Firstly, we applied the double-robust inverse-probability-weighted regression adjustment (IPWRA) approach, which has the advantage over conventional propensity score methods as it makes use of all the available information. Secondly, from the treatment effects estimation, both the relative and absolute effects of treatment on survival time were estimated. Past studies have focused on relative measures that are helpful since they provide relative risks or advantage information of alternative treatments. However, absolute measures of treatment effects complement relative measures in that they not only provide quantifiable and meaningful information for both physician and patients when they choose between alternative treatments, but also provide a useful basis for the economic evaluations of different RRT. Thirdly, we have information for all Swedish ESRD patients that ensures the statistical power and generalization of our study. 

## 2. Materials and Methods 

### 2.1. Data Sources

The data of this study were based on the Swedish Renal Registry (SRR) [13] which is linked to the Register of the Total Population (RTB) [14], the Scandia transplant database [15], and the Longitudinal Integration Database for Health Insurance and Labor Market Studies (LISA by Swedish acronym) [16], using a unique national personal identification number. The SRR is a high-quality registry that records patients’ baseline characteristics, treatment modality and date and cause of death information. The RTB includes marital status and citizenship information while LISA includes socioeconomic status-related data (e.g., income and education) up to 10 years before and after the RRT start. The Scandia transplant database provides information on waitlisting. For a subsample of the study population, we also had detailed healthcare utilization from the regional healthcare utilization databases of Region Skåne and Stockholm County Council, two healthcare administrative areas in Sweden covering around 1/3 of the Swedish population. 

### 2.2. Patients Characteristics

The study included all 16,943 adult ESRD patients who started RRT between 1 January 1995, and 31 December 2012. Patients were excluded according to the following criteria: (1) the current treatment modality was unknown (six patients, 0.04%); (2) recovered or died within 91 days of the start of dialysis (1819 patients, 10.74%); (3) a lack of waitlist information (434 patients, 2.56%); and (4) have missing information for other important factors (i.e., income (259 patients, 1.53%), education (357 patients, 2.11%), marital status (86 patients, 0.51%), and KTx center (105 patients, 0.62%)). Therefore, the final sample included 13,877 adult patients on RRT out of which 2676 were on the waitlist for transplantation. Each patient was followed to death or the end of the study (June 2015). 

Baseline data included demographics (age, sex, year of first RRT, citizenship (Swedish vs. non-Swedish), KTx center, and marital status), socioeconomic status (income and education), clinical characteristics (blood type, comorbidities, and primary renal disease). This information was collected before the start of RRT. Income was defined as equivalized individual disposable income and divided into quintiles [17]. Education was categorized, according to the Swedish educational system, into mandatory education (≤9 years), secondary education (>9–12 years), and higher education (>12 years) [17]. Primary renal diseases were grouped into seven categories: glomerulonephritis, adult polycystic kidney disease, diabetes mellitus, hypertension, pyelonephritis, unspecified kidney disease, and others (for all other renal diagnoses). We re-categorized the comorbidities registered in SRR as hypertension, diabetes mellitus, cancer (blood-, skin-, and other cancer) and cardiovascular disease (cerebrovascular-, peripheral vascular-, ischemic-, and other cardiovascular disease), due to a low prevalence in certain groups.

### 2.3. Exposures and Outcomes

Patients on the waiting list, not receiving a renal transplant, were assigned to the dialysis group that includes hemodialysis and peritoneal dialysis. Patients were included in the kidney transplantation group if they got KTx during the follow-up period. We adopted an intension-to-treat approach, which is common in previously published articles. Patients with a failed renal transplant were not censored in the intention to treat analyses, as this is relevant for the treatment effect of renal transplantation. If censoring at a time of lost graft, it would overestimate the benefit of renal transplantation [8].

The main outcome of the study was survival time after RRT was started. Time to death was defined from the start date of the RRT until the date of death. Patients who did not die were censored at the end of study (June 2015) in all statistical analyses.

### 2.4. Statistical Analyses

This study estimated the POMs and the ATEs. The POM for KTx refers to the average survival time if all the patients get KTx (Y1) while the POM for dialysis refers to the average survival time if all the patients get dialysis (Y0). The ATE is the difference of the average survival time between KTx and dialysis over the whole sample [18]. 

The difficulty in estimating the ATEs is that we only observe Y1 or Y0 (getting KTx or not getting KTx) for each patient in the observational data. When the treatment is assigned randomly in RCTs, the randomization ensures that the POMs are independent of the factors influencing treatment assignment. In observational studies, the treatment is not randomly assigned and the conditional independence assumption is needed in order to estimate the ATEs. The conditional independence assumption says that there is no bias if the outcome is independent of the factors influencing treatment assignment after conditioning on a sufficient number of covariates. 

We used the IPWRA estimator that uses weighted regression coefficients to calculate the predicted outcome for each individual in each treatment and then the average of predicted outcomes for each treatment, where the weights are the estimated inverse probabilities of having each treatment. This means that an observation is given a higher weight, the more unlikely their treatment assignment. The first step estimates the probability of treatment using a logit regression model including variables that affect treatment assignment and outcomes at baseline. There is a lack of consensus in the literature as to which variables should be included in the propensity score model [19]. We included as many pre-treatment covariates related to treatment assignment as possible. The included covariates were customary in previously published articles related to this topic and considered to be conceivably related to both survival and the choice of modality [8,20,21,22]. The second step uses regression adjustment analysis, with weights provided by the inverse of the estimated probability that a patient received a treatment modality [23]. The weights do not bias the regression adjustment (RA) estimator if the treatment model is wrongly specified, providing the outcome model is correct. Similarly, the weights adjust the RA estimator if the treatment model is appropriate but the outcome model is wrongly specified, i.e., the IPWRA is a so-called double-robust method [23]. We assessed the goodness-of-fit and specification of the treatment model by using the Hosmer–Lemeshow c statistic and the Pregibon link test to study if the double robust property holds [24,25]. The Hosmer–Lemeshow c statistic evaluates whether the difference between observed and predicted values of the response variable are significant. The failure to reject the null hypothesis of no differences is a signal of good model calibration [24]. The Pregibon link test estimates the treatment effects equation with the linear predicted value and the squared linear predicted value as the only two explanatory variables (besides a constant). If the treatment equation is correctly specified, the coefficient of the squared linear predicted value should be non-significant [25]. The Akaike’s information criterion (AIC) approach was used to compare the fit of the outcome models using different distributions where a smaller AIC statistic suggests a better fit [26].

We used the standardized differences method to assess the balance of baseline covariates between the KTx group and the dialysis group in the sample before and after we weighted them by the inverse probability of treatment [27,28]. Compared with traditional significance testing, standardized differences are not as sensitive to sample size and are useful in identifying meaningful differences. Typically, a standardized difference greater than 0.1 is considered meaningful [29]. We also did a formal over-identification test for covariate balance after weighting. Curtis [12] noted that we should pay careful attention to contraindication to the treatments of interest. In the case where the likelihood of receiving a treatment is zero, the inverse probability-weighted estimation is not an appropriate approach. We therefore evaluated the estimated probabilities to ensure there are no very large (close to 1) or very small (close to 0) ones. The overlap assumption (i.e., each patient had a positive probability of getting each treatment) was assessed using an overlap plot. 

After estimating the POMs and ATEs, we also conducted a separate estimation of the POMs and ATEs for sex and then compared the difference of ATEs between men and women using the t-test. Statistical significance was assumed for *p*-values < 0.05. All statistical analyses were performed using Stata software, version 14.0 (College Station, Texas, USA). This study has been approved by Lund Regional Ethical Review Board (Dnr: 2014/144). 

### 2.5. Sensitivity Analysis

The main analysis included only patients on the waitlist in order to minimize the selection bias. However, some previous studies also focused on the general dialysis patients. To compare our results with the new method to those results from previous studies, we also created a study population of all patients who started RRT between the years 1995 and 2012, irrespective of waitlisted status. We can still check the covariates’ balance between the two groups after weighting even though we focused on the general dialysis patients. If balance is also achieved between KTx patients and the general dialysis patients, we can obtain reliable results to compare with the results from previous studies.

The Charlson comorbidity index (CCI) is a simple and valid method of estimating the risk of death from comorbid diseases for use in longitudinal studies. This takes into account both the number and the seriousness of comorbid diseases and can be calculated by using the diagnoses before the start of RRT [30]. In the main analysis, we only controlled for those comorbidities registered in the SRR and we therefore re-ran the analyses including the CCI for both waitlisted and the full patient sample. However, detailed information on prior diagnoses needed for calculating the CCI was only available for a subsample of patients (two healthcare administrative areas) and therefore the main analysis for this sample was re-run for comparison reasons. 

In our study, patients who never received a transplantation were assigned to the dialysis group. While there was a risk that the “sickest dialysis patients” died before a suitable donor became available, patients in the KTx group, by definition, survived this period. Consequently, this might introduce what is known as “immortal time bias” [31]. Even though limiting patients on the waitlist can reduce the immortal time bias, it could still exist if dialysis patients on the waiting list are more likely to die early compared to those that get a transplant. Therefore, we performed a landmark analysis in a sensitivity analysis to study if immortal bias could be a concern in this study. The most important thing in landmark analysis was to choose a clinically relevant landmark, that is, a point on the time axis at which we could classify patients into those who had already had KTx and those who were still on dialysis at that time [31]. In this sensitivity analysis, we used both mean and median waiting times (1.4 years and 1.1 years) for KTx as the landmarks to see if the main results are stable.

## 3. Results

### 3.1. Descriptive Analysis and Model Assessment

Two thousand six hundred and seventy-six (2676) adult patients, of whom 2151 (80.4%) received a KTx, were observed during the study period. Table 1 shows the baseline characteristics and standardized differences before and after weighting in the dialysis and kidney transplantation groups for the patients on the waiting list. Before weighting, the patients on dialysis were older, less educated, had lower income, and had more comorbidities. The weighting reduced the standardized differences compared to the unweighted data to an acceptable range (<|0.10|). Moreover, the over-identification test for covariate balance indicated that the weighted groups were balanced (*p* = 0.56) and we concluded that the balance of covariates between the KTx group and the dialysis group was satisfying. 

**Figure 1:**
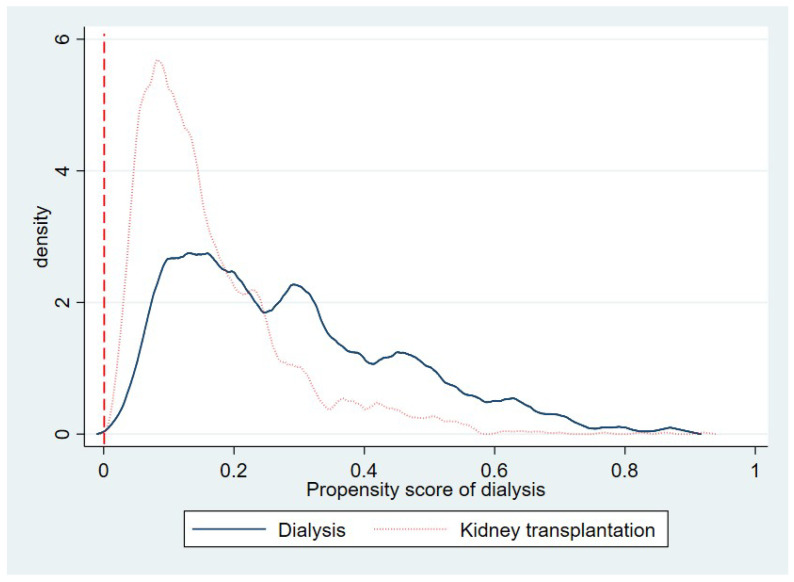
Overlap plot by treatment groups for baseline analysis—the probability of being in the dialysis group.

Figure 1 is the overlap plot that displays the estimated density of the predicted probabilities of getting KTx or dialysis. The plot indicates that the probability mass is not too close to 0 or 1, and the two estimated densities have most of their respective masses in regions in which they overlap each other. Thus, there is no evidence that the overlap assumption is violated. 

We failed to reject the null hypothesis of no difference between the observed and predicted values of the response variable (getting KTx) using the Hosmer–Lemeshow c statistic (*p* = 0.46). The Pregibon specification test suggested that the treatment equation was well specified as the parameter estimate of the squared of the predicted values was non-significant (*p* = 0.11). Thus, the treatment model was considered appropriate and the double robust property of the IPWRA estimator appears to hold. The AIC approach suggested that the Weibull distribution is the most suitable distribution for our outcome model (AIC = 4062.2).

### 3.2. Average Treatment Effect for Waitlisted Patients and Subgroup Analysis by Sex

Table 2 shows the estimated ATE on the survival time for waitlisted patients and the ATE by sex. The estimated average survival time would be 23.1 years if all patients received a renal transplant, 13.8 years (1.48 times) longer than if all patients received dialysis. In the subgroup analysis by sex, the estimated average survival time would be 22.9 years if all men got a renal transplant, 14.4 years (1.71 times) longer than if all men would get dialysis. Similarly, the estimated survival time was 24.2 years if all women received a renal transplant, 13.9 years (1.34 times) longer than if all women received dialysis. The difference in ATE between sexes is not significant (*p* = 0.90).

**Figure 2:**
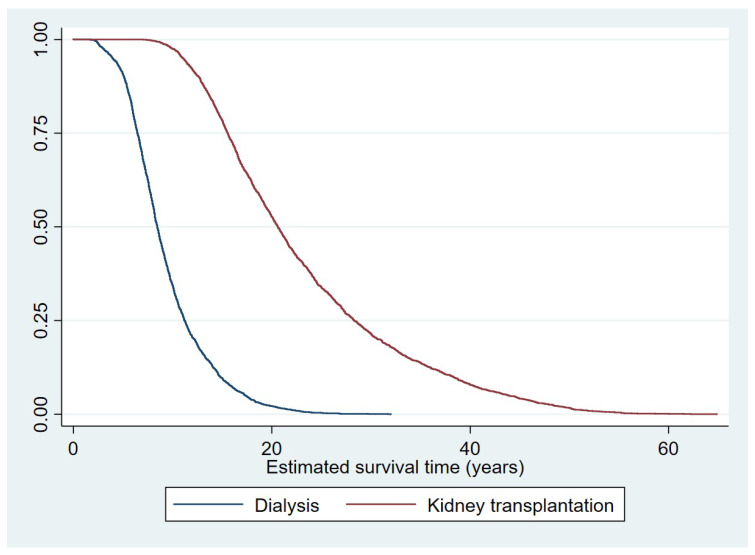
Kaplan–Meier estimated survival curve by treatment groups for main analysis.

Figure 2 shows the estimated survival time as a Kaplan–Meier survival curve. These are the predicted survival times (POM of each patient) in each treatment from the IPWRA estimations, i.e., the weighted adjusted survival time on the x axis and the proportion of the population alive on the y axis.

### 3.3. Sensitivity Analyses

#### 3.3.1. Average Treatment Effect for All RRT Patients and Subgroup Analysis by Sex

Table 3 shows the ATE on the survival time for all RRT patients (i.e., not only patients on the waiting list) and ATE by sex. The average survival time was estimated to be 15.5 years if all patients on RRT received KTx, which was 11.1 years (2.51 times) longer compared to if all patients on RRT received dialysis. In the subgroup analysis for both men and women, the average survival time was estimated to be around 2.5 times longer if patients got a renal transplant than if patients got dialysis. Although women had longer survival times than men in both the KTx and dialysis treatments, there was no difference in the ATE over sex (*p* = 0.86).

#### 3.3.2. Average Treatment Effect When Controlling for the Charlson Comorbidity Index

Table 4 show the ATE for the sub-sample living in Region Skåne and Stockholm County Council controlling for the CCI as well for both waitlisted and all RRT patients. The estimated survival times did not change when the CCI was added. 

#### 3.3.3. Average Treatment Effect for Waitlisted Patients When Controlling for Immortal Time Bias Using Landmark Analysis

After using the landmark analysis controlling for immortal time bias, we found that the estimated survival time for dialysis patients was (slightly) longer (12.0 years and 11.2 years for the mean and median waiting times, respectively) compared to the main analysis (9.3 years). This translated into a smaller ATE (9.1 years and 9.9 years for mean and median waiting time, respectively) compared to the main analysis (13.8 years).

## 4. Discussion

We assessed the treatment effect of renal replacement therapy (RRT) on survival time using the double robust inverse-probability-weighted regression adjustment approach. Our results showed that renal transplantation increases survival time in Swedish patients on RRT. For patients on the waitlist, the (absolute) average survival advantage of transplantation is almost 14 years compared to dialysis after controlling for selection into treatment. Using all patients on RRT resulted in similar survival benefit although the estimated survival times were much shorter. This indicates that KTx can be expected to give a substantial survival advantage also for patients not being currently considered for transplantation. No difference between men and women in terms of average survival benefit of KTx could be found.

Previous studies have usually used the (relative) hazard ratio as the effect measure when comparing mortality between different treatments. Comparing our results directly to prior studies is therefore not feasible. While our findings confirm the results of previous studies in terms of the relative survival advantage of renal transplantation [3,4,8], we also provide new information about the absolute survival advantage which is important, for example, in economic evaluations of interventions, where often cost per live-year gained is of interest. 

Compared with previous studies, our study took account of both the selection bias to different treatment modalities and the selection bias was related to waitlisting and applied advanced statistical analyses comparing KTx with dialysis. Bayat et al. [8] compared the survival of patients with renal transplants with the general dialysis patients in a French region and focused on elderly patients. They used an estimated propensity score to control for non-random treatment assignment to the waitlist for KTx and showed that KTx had longer survival. However, the mortality of general dialysis patients was higher than dialysis patients on the waitlist because of selection bias to waitlisting [8]. Our study compared KTx patients with waitlisted dialysis patients and used a propensity score weighting method, which to some extent reduced the second selection bias. We also compared KTx patients with general dialysis patients and found that general dialysis patients had shorter survival compared to KTx patients which was consistent with the study of Bayat et al. [8]. 

In the subgroup analysis, women had longer survival time than men in both KTx and dialysis therapy both for patients on the waitlist and for all RRT patients. The higher potential outcome means for women might reflect their longer life expectancy in general. However, there was no difference in the average treatment effects (ATEs) between men and women. Our previous study showed that women had the same chance to access a kidney transplant as men [17]. It thus seems that there is no gender inequality neither in access to kidney transplantation or in the survival advantage of KTx in Sweden during the study period.

The comorbidities registered in the Swedish Renal Registry (SRR) cannot be included in any validated comorbidity index. Therefore, we included the CCI based on healthcare utilization data available for a subsample. Our sensitivity analysis showed that the inclusion of the CCI did not change the results compared to the baseline estimation using registered comorbidities in the SRR. This indicates that our baseline results are valid with regard to comorbidities.

Immortal bias may exist since the dialysis patients may have died before they could access KTx. However, we restricted the sample to patients on the waitlist, which excluded patients who died shortly after starting RRT, and the immortal bias has thus probably been reduced. We further applied a landmark analysis to test the existence of the immortal time bias, which resulted in a smaller survival benefit of transplantation although still substantially better than dialysis. Thus, our baseline survival benefit estimates could potentially be upward biased.

The main strength of the current study lies in the use and linkage of several high-quality Swedish register databases with almost 100% coverage of Swedish RRT patients and a data-reporting incidence of 95% [32]. Moreover, in contrast with previous studies from the USA and France [4,8], our data enabled taking into account all the major primary renal diseases, socioeconomic status variables and comorbidities. Based on the rich and high-quality information in the database, the RRT treatment effects were estimated by using the IPWRA approach, which allowed us to estimate what the survival time would be if all patients on dialysis received renal transplants or vice versa. Propensity score methods are often applied incorrectly when estimating the effect of treatment on time-to-event outcome. Common errors include the use of inappropriate statistical tests and the failure to assess correctly if the specified propensity score model has induced acceptable balance in baseline covariates between treatment and control groups [19]. Compared with previous studies, we did not only test the assumptions when using the propensity score method, but did also check the baseline balance after weighting and model assessments to see if the double robust property of IPWRA holds, which increases the credibility of our results. Even though the hazard ratio is a popular effect measure when comparing the mortalities of different treatments, it is mainly useful when the treatment enters linearly and the distribution of the outcome has a proportional hazards form [33]. However, when using ATEs as an effect measure, neither linearity in treatment nor a proportional hazards form is required. Moreover, the ATE measures the effect in the same time units as the outcome instead of in relative conditional probabilities and the ATE is much easier to explain, also to non-technical audiences [34].

The main limitation of our study was that although we controlled for observable variables to reduce the selection bias, we can never rule out that an unobservable factor may still influence the results. However, unobservable variables are only a problem if they are correlated with both treatment selection and outcome measure. If this is the case, but the unobservable variable is highly correlated with the propensity score/controlled observable variables, the results with respect to treatment effects should not be greatly affected. If this is not the case, the results may be influenced. However, the linked database provided a very rich source of information, and we made extensive use of the information available in it, therefore trying to minimize this risk. Although this set of variables is broad, some important prognostic factors which could have an impact on the choice of RRT were not available, such as BMI, parathyroid hormone level and the availability of kidneys for transplantation [35]. However, the controlled comorbidities and the CCI in our study may capture part of the effects of these prognostic factors.

## 5. Conclusions

The present study shows the survival advantage of renal transplantation compared to dialysis in Swedish patients with RRT, both in absolute and relative terms, and both among those selected to the waiting list and those currently not listed. The results are strong incentives for an increased frequency of renal transplantations. The treatment effects estimation approach is new in the context of RRT. Our attempt is an effort to establish a direction for future research estimating the treatment effects in this area. Further studies are needed to assess the approach and to quantify the effect of renal transplantation cost and quality of life.

## Figures and Tables

**Table 1:** Baseline characteristics before and after weighting in the dialysis and kidney transplantation groups for the patients on the waitlist (*n* = 2676).

Baseline Variable	Before Weighting			After Weighting		
	Dialysis, *n* = 525	KTx, *n* = 2151	Standardized Difference ^#^	Dialysis, *n* = 1325.1 *	KTx, *n* = 1350.9	Standardized Difference
Age at start RRT, years (ref = 18–39), %						
40–49	22.9	22.7	−0.003	17.7	17.6	−0.017
50–59	32.4	35.5	0.065	21.9	22.9	−0.045
60+	35.6	25.2	−0.229	23.0	18.8	0.007
Men, %	67.0	65.9	−0.025	22.1	22.5	−0.063
Year of first RRT (ref = 1995), %						
1996	5.1	6.2	0.045	4.9	5.8	−0.066
1997	5.1	6.2	0.045	4.9	5.8	0.003
1998	6.1	5.7	−0.016	5.7	5.4	−0.031
1999	6.5	7.0	0.022	6.1	6.5	−0.000
2000	5.3	5.9	0.023	5.1	5.5	−0.045
2001	5.1	5.8	0.029	4.9	5.5	−0.008
2002	4.0	6.0	0.092	3.8	5.6	0.017
2003	4.0	8.2	0.177	3.8	7.6	0.019
2004	8.0	6.3	−0.067	7.4	5.9	−0.010
2005	4.4	4.6	0.013	4.2	4.4	−0.012
2006	5.0	6.0	0.046	4.7	5.6	0.061
2007	5.0	4.2	−0.037	4.7	4.0	−0.032
2008	5.3	2.6	−0.140	5.1	2.5	0.012
2009	6.3	1.3	−0.263	5.9	1.3	0.022
2010	5.1	5.4	0.011	4.9	5.1	0.054
2011	5.9	6.0	0.004	5.6	5.6	−0.0.13
2012	7.0	6.6	−0.020	6.6	6.1	0.016
Education (ref = mandatory), %						
Secondary school	46.1	45.4	−0.014	24.9	24.8	−0.026
Higher education	19.2	24.4	0.125	15.6	18.5	0.014
Disposable income (ref = quintile 1), %						
Quintile 2	15.6	15.8	0.005	13.2	13.3	0.047
Quintile 3	17.1	14.5	−0.072	14.2	12.4	−0.012
Quintile 4	23.2	20.6	−0.064	17.9	16.4	0.004
Quintile 5	25.7	30.4	0.104	19.1	21.2	−0.027
Marital status (ref = married), %						
Single	26.9	29.8	0.064	19.7	20.9	−0.039
Divorced	20.8	16.4	−0.112	16.5	13.7	0.026
Widowed	4.2	2.9	−0.071	4.0	2.8	−0.014
Citizenship (ref = non-Swedish), %						
Swedish	85.0	86.2	0.037	12.8	11.9	−0.010
KTx center ^§^ (ref = no KTx center), %						
KTx center	55.6	50.7	−0.099	24.7	25.0	0.019
Primary renal disease (ref = APKD), %						
Diabetic nephropathy	34.3	16.2	−0.426	22.6	13.6	−0.007
Glomerulonephritis	18.7	28.0	0.223	15.2	20.2	−0.034
Hypertension	9.9	7.2	−0.098	8.9	6.6	0.009
Pyelonephritis	2.1	4.1	0.115	2.1	3.9	0.060
Unspecified kidney disease	6.9	8.4	0.059	6.4	7.7	−0.005
Other	18.1	21.0	0.072	14.8	16.6	0.012
Comorbidities, %						
Hypertension	69.5	69.4	−0.003	21.2	21.3	−0.026
Diabetes mellitus	33.7	17.3	−0.382	22.4	14.3	−0.034
Cardiovascular disease	23.2	14.3	−0.230	17.9	12.3	−0.008
Cancer	3.6	3.3	−0.020	3.5	3.1	−0.032
Blood type (ref = O), %						
A	32.8	45.8	0.269	22.1	24.8	−0.026
B	12.8	12.4	−0.012	11.2	10.8	0.014
AB	3.0	5.7	0.131	3.0	5.4	−0.011

^#^ Standardized differences are computed as: $\frac{{\bar{x}}_{treated}-{\bar{x}}_{nontreated}}{\sqrt{\frac{s_{treated}^{2}+s_{nontreated}^{2}}{2}}}$ where ${\bar{x}}_{treated}$ and ${\bar{x}}_{nontreated}$ denote the sample mean of the covariate in the treated and non-treated subjects, respectively. $s_{treated}^{2}$ and $s_{nontreated}^{2}$ denote the sample variance of the covariate in the treated and non-treated subjects. Standardized differences > |0.10| are generally considered meaningful. RRT = renal replacement therapy; ref = reference group; KTx = kidney transplantation; APKD = adult polycystic kidney disease. * Weighted sample size. ^§^ Whether patient’s home county has a Tx center. Equivalized individual disposable income was divided into quintiles, where quintile 1 represents the most disadvantaged and quintile 5 the most advantaged. Categorical variables are presented as percent of the total.

**Table 2:** Average treatment effect of the treatment on the survival time for patients on the waitlist and subgroup analysis by sex (*n* = 2676).

	Main Analysis	Subgroup Analysis by Sex		
	Coef. (95% CI)	Coef. (95% CI): Men	Coef. (95% CI): Women	Test ATE: –Women + Men = 0 (*p*)
ATE				0.896
KTx vs. Dialysis	13.8 (11.4–16.2)	14.4 (11.3–17.6)	13.9 (6.2–21.5)	
POM				
KTx	23.1 (21.2–25.0)	22.9 (20.8–25.0)	24.2 (19.0–29.5)	
Dialysis	9.3 (7.8–10.8)	8.5 (6.6–10.3)	10.4 (3.0–17.8)	

CI = confidence interval; KTx = kidney transplantation; ATE = average treatment effect; POM = potential outcome mean.

**Table 3:** Sensitive analysis: average treatment effect for all RRT patients and subgroup analysis by sex (*n* = 13,877).

	Main Analysis	Subgroup Analysis by Sex		
	Coef. (95% CI)	Coef. (95% CI): Men	Coef. (95% CI): Women	Test ATE: –Women + Men = 0 (*p*)
ATE				0.861
KTx vs. Dialysis	11.1 (9.6–12.6)	11.2 (9.2–13.1)	11.6 (6.7–16.5)	
POM				
KTx	15.5 (14.0–17.0)	15.5 (13.4–17.6)	16.2 (12.1–20.3)	
Dialysis	4.4 (4.3–4.6)	4.3 (4.2–4.5)	4.6 (4.3–4.9)	

CI = confidence interval; KTx = kidney transplantation; ATE = average treatment effect; POM = potential outcome mean.

**Table 4:** Sensitive analysis: average treatment effect for Region Skåne and Stockholm using comorbidities registered in the SRR and while controlling for the Charlson comorbidity index.

	Using Registered Comorbidities Coef. (95% CI)	Charlson Comorbidity Index Coef. (95% CI)
For patients on the waitlist (*n* = 1129)		
ATE		
KTx vs. dialysis	12.0 (6.8–17.3)	11.9 (6.8–17.1)
POM		
KTx	25.0 (20.9–29.0)	24.7 (20.7–28.7)
Dialysis	12.9 (9.5–16.4)	12.8 (9.4–16.2)
For all RRT patients (*n* = 4519)		
ATE		
KTx vs. dialysis	12.4 (9.4–15.4)	12.3 (9.0–15.5)
POM		
KTx	17.5 (14.5–20.5)	17.4 (14.2–20.6)
Dialysis	5.2 (4.9–5.4)	5.2 (4.9–5.5)

CI = confidence interval; KTx = kidney transplantation; ATE = average treatment effect; POM = potential outcome mean.
